# Combined Effect of Silybin and Sodium Acetylated Hyaluronate on UV‐Induced Photoaging

**DOI:** 10.1111/srt.70382

**Published:** 2026-09-12

**Authors:** Sen Hou, Hanliang Song, Jianying Zhang, Anzhang Li

**Affiliations:** ^1^ Guyu Biotechnology Group Co., Ltd Guangzhou Guangdong China; ^2^ Guangzhou Qingnang Biotechnology Co., Ltd Guangzhou Guangdong China; ^3^ Guangzhou Zhongkejian Technology Testing Co., Ltd Guangzhou Guangdong China

**Keywords:** collagen, photoaging, ROS, silybin, sodium acetylated hyaluronate

## Abstract

**Background:**

Exposure to solar ultraviolet (UV) radiation is a major cause of skin photoaging, which is characterized by decreased collagen levels in the dermis. Both silybin and sodium acetylated hyaluronate (HA) are potential anti‐aging compounds. However, when used alone, both agents show limited anti‐aging efficacy. This study aimed to examine the combined effects of silybin and HA on UV‐induced skin photoaging and elucidate the underlying mechanisms.

**Methods:**

In this study, cytotoxicities of silybin and HA were assessed across a range of concentrations, both with and without UV exposure. We quantified mRNA levels of matrix metalloproteinase‐1 (MMP‐1), collagen type I (Col I), and collagen type III (Col III) after silybin and HA treatment to study their collagen synthesis promotion and degradation inhibition effects. Reducibility and reactive oxygen species (ROS) scavenging activity were also characterized for each compound. Collagen protein content was then measured after individual and combined treatments.

**Results:**

Silybin showed obvious cytotoxicity when its concentration reached 0.01%, while HA remained largely non‐toxic even at concentrations of 1%. UV irradiation slightly increased the cytotoxicity of both silybin and HA. Notably, silybin effectively inhibited collagen degradation by downregulating MMP‐1 mRNA expression, although it failed to upregulate Col I and III mRNA levels. In contrast, HA did not inhibit, and at higher concentrations slightly elevated, MMP‐1 mRNA expression, while it was efficient in promoting Col I and III mRNA expression. Therefore, combined treatment with silybin and HA led to the downregulation of MMP‐1 mRNA levels and upregulation of Col I and III mRNA levels. Silybin also showed high reducibility and therefore provided strong ROS scavenging capacity. As MMP‐1 synthesis is primarily induced by ROS, silybin exhibited strong MMP‐1 inhibition. Meanwhile, HA showed weak ROS scavenging capacity owing to its low reducibility. Nevertheless, the combination of silybin and HA inhibited collagen degradation (primarily via silybin) and promoted Col I and III mRNA expression and protein synthesis (primarily via HA), thereby increasing overall collagen protein levels.

**Conclusions:**

This study reveals the complementary mechanisms through which silybin and HA collectively increase dermal collagen levels—silybin by protecting existing collagen from degradation and HA by promoting collagen synthesis—guiding their combined use in cosmetics to protect the skin from UV‐induced photoaging.

## 1 Introduction

Solar radiation is among the primary contributors to skin aging [1]. Ultraviolet A (UVA) radiation (320–400 nm), which accounts for approximately 95% of the UV radiation that penetrates the atmosphere, is the most damaging component of solar light [2]. UVA can penetrate deeply into the skin's dermis, decreasing collagen production in fibroblasts and increasing collagen degradation. Owing to the decrease in collagen, the extracellular matrix (ECM) of the dermis loses structural support, leading to the appearance of wrinkles and visual signs of skin aging [3]. Numerous compounds have been reported to counteract skin photoaging by increasing collagen levels in the skin [4]. These agents can be classified into two categories based on their mechanism of action: those that promote collagen synthesis and those that inhibit collagen degradation. For instance, silybin has been shown to inhibit collagen degradation by downregulating the expression of matrix metalloproteinases (MMPs) [5]. Meanwhile, evidence shows that sodium acetylated hyaluronate (HA) can enhance collagen synthesis. The combined use of silybin and HA shows great potential for its possible complementary effect in promoting collagen content in the skin.

Fibroblasts synthesize collagens, proteoglycans, and elastin [6]. Type I and type III collagens are the predominant forms of collagen found in the dermis. Photoaging induced by UV radiation is caused by both the inhibition of ECM synthesis and the stimulation of ECM degradation. This imbalance contributes to dermal thinning and clinical manifestations such as wrinkle formation and other visual signs of aging [7]. MMPs show accumulation during aging. Since their levels are upregulated by reactive oxygen species (ROS) generated in response to UV exposure [8]. MMP‐1 is the most efficient protease for cleaving the triple helix structure of type I and type III collagen fibers, and the resulting fragments are subsequently degraded by MMP‐3 and MMP‐9 [9]. Consequently, photoaged fibroblasts exhibit a reduced capacity for collagen synthesis partly due to the accumulation of prostaglandin E2 (PGE2), an inhibitor of collagen production [10].

Silymarin, an extract derived from the seeds of *Silybum marianum* (milk thistle), has been shown to effectively prevent UV‐induced skin damage. Notably, *S. marianum* has been used in European herbal medicine for centuries [11]. The primary polyphenolic constituent of silymarin is the flavonolignan silybin, which has been found to suppress the ROS production in UV‐irradiated HaCaT keratinocytes [12]. Since ROS is a major inducer of MMPs, which degrade dermal collagen, the ability of silybin to protect collagen from degradation in the skin is primarily attributed to its inhibitory effect on MMP expression.

Hyaluronic acid is a glycosaminoglycan composed of repeating disaccharide units of D‐glucuronic acid and N‐acetyl‐D‐glucosamine [13]. Due to its efficacy in promoting skin hydration, hyaluronic acid is one of the most widely recognized active ingredients in cosmetic products. Hyaluronic acids with intermediate (approximately 300 kDa) and low (20–50 kDa) molecular weights have been reported to penetrate deeply into the skin [14]. Acetylated hyaluronic acid, specifically HA, shows increased resistance to hyaluronidase degradation and enhanced skin penetration [15]. HA is also believed to promote cell regeneration and stimulate collagen production through its nutricosmetic effects [16, 17, 18].

In this study, we studied the potential combined effect of silybin and HA in increasing collagen content in UV‐induced photoaged fibroblasts. We tested the joint cytotoxicity of silybin and HA. Furthermore, we evaluated their combined ability to inhibit MMP mRNA expression and promote Col I and III mRNA levels. In addition, we examined the mechanism underlying their actions by assessing their ROS‐scavenging activity and antioxidant capacity via ABTS assays. Finally, we measured their combined effect on collagen protein synthesis in fibroblasts.

## 2 Materials and Methods

### 2.1 Materials

HA with an average molecular weight of 10000 Da was purchased from Sichuan Liyuan Bo Technology Co. Ltd., China. The degree of acetylation was more than 95% according to the production manual. Silybin with a purity of 98% was purchased from StarHealth Co. Ltd., China. Human skin fibroblast (HSF) cells were purchased from Fenghui Biotechnology Co., Ltd., China. HSF cells were routinely cultured in Dulbecco's Modified Eagle Medium (DMEM) containing 10% fetal bovine serum (FBS, Gibco, USA).

### 2.2 Cell Viability Assay

Before the experiment, HSF cells were digested using 0.25% Trypsin‐EDTA. The cells were then seeded in 96‐well plates at a density of 0.8 × 10^5^ cells/cm^2^ and cultured for 24 h. The cells were incubated with DMEM containing silybin, HA, or a mixture of silybin and HA for 24 h, and the concentration‐dependent cytotoxicity was tested via the MTT assay. The tested concentrations of silybin were 0.00005%, 0.0001%, 0.0005%, 0.0001%, 0.005%, 0.001%, and 0.05% (w/w), respectively. The concentrations of HA were 0.001%, 0.005%, 0.01%, 0.05%, 0.1%, 0.5%, and 1% (w/w), respectively. The concentrations of silybin and HA in the mixtures were 0.00001% (silybin)+0.001% (HA), 0.00005% (silybin)+0.005% (HA), 0.0001% (silybin)+0.01% (HA), 0.0005% (silybin)+0.05% (HA), 0.001% (silybin)+0.1% (HA), 0.005% (silybin)+0.5% (HA), and 0.01% (silybin)+1% (HA) (w/w), respectively. Untreated cells served as controls. For the MTT assay, the medium in each well was replaced with 100 µL of DMEM containing 0.5 mg/mL MTT. Then, the cells were incubated in this medium at 37°C for 4 h. After incubation, the medium in each well was replaced with 150 µL of DMSO solution. The optical density (OD) values were measured at a wavelength of 490 nm using a microplate reader (Epoch2, BioTek, USA). In order to understand the influence of UV irradiation, the same cytotoxicity experiments were also repeated following UVA irradiation (20 J/cm^2^). All measurements were repeated three times. The results were presented as the average value ± standard deviation.

### 2.3 Quantitative Real‐Time PCR Assay

The mRNA expression of MMP‐1, Col I and Col III was measured via quantitative real‐time PCR (qPCR). HSF cells were seeded in 12‐well plates at a density of 1 × 10^5^ cells/cm^2^ and cultured for 24 h. Then, the cells were exposed to PBS containing different concentrations of silybin, HA, or a mixture of silybin and HA, followed by 20 J/cm^2^ UVA irradiation. Subsequently, the cells were incubated with DMEM containing the same concentration of silybin, HA, or their mixture and cultured for an additional 24 h. The tested concentrations of silybin were 0.0001%, 0.005%, and 0.001% (w/w), respectively. The concentrations of HA were 0.01%, 0.05%, and 0.1% (w/w), respectively. The concentrations of silybin and HA in the mixtures were 0.0001% (silybin)+0.01% (HA), 0.005% (silybin)+0.05% (HA), and 0.001% (silybin)+0.1% (HA) (w/w), respectively. After treatment, the cells were rinsed with PBS and lysed to extract total RNA using the RNA‐easy Isolation Reagent (Vazyme Biotech, China) according to the manufacturer's instructions. Then, the cDNA was produced using the HiScript II Q RT SuperMix for qPCR (Vazyme Biotech, China). Finally, qPCR was performed using the ChamQ Universal SYBR qPCR Master Mix (Vazyme Biotech, China) on a CFX Opus 96 real‐time PCR system (Bio‐Rad, USA) equipped with Bio‐Rad CFX Maestro software. The relative expression of the target genes was normalized based on the expression of the internal control β‐actin. Untreated cells served as the blank group. Cells treated with UV irradiation alone served as the negative control group. All measurements were repeated three times. The results were presented as the average value ± standard deviation. All primer sequences used for qPCR are shown in Table 1.

**TABLE 1:** Primer sequences.

Gene	Primer sequence
MMP1‐F	5’‐ATTCTACTGATATCGGGGCTT‐3’
MMP1‐R	5’‐ATGTCCTTGGGGTATCCGTGTAG‐3’
COLIA1‐F	5’‐CGTCATCGCACAACACCTTG‐3’
COLIA1‐R	5’‐AGTCACTACTGACAACGCCC‐3’
COLIIIA1‐F	5’‐CTTCTCTCCAGCCGAGCTTC‐3’
COLIIIA1‐R	5’‐AATGTTTCCTACCGTGCAACC‐3’
Hum‐*β*‐actin‐F	5’‐GTCCACCTTCCAGCAGATGT‐3’
Hum‐*β*‐actin‐R	5’‐GTCACCTTCACCGTTCCAGT‐3’

### 2.4 ROS Scavenging Assay

HSF cells were seeded in 96‐well plates at a density of 0.8 × 10^5^ cells/cm^2^ and cultured for 24 h. The cell culture medium was removed, and the cells were incubated with 100 µL of DMEM containing silybin, HA, or a mixture of the two for 24 h. The tested concentrations of silybin were 0.0001%, 0.005%, and 0.001% (w/w), respectively. The concentrations of HA were 0.01%, 0.05%, and 0.1% (w/w), respectively. The concentrations of silybin and HA in the mixtures were 0.0001% (silybin)+0.01% (HA), 0.005% (silybin)+0.05% (HA), and 0.001% (silybin)+0.1% (HA) (w/w), respectively. After incubation, H_2_O_2_ was added to each well (final concentration, 250 µM). After 1 h, the cell culture medium was removed, and the ROS levels were measured with a ROS Assay Kit (Beyotime, China) according to the manufacturer's instructions. The fluorescence intensity was measured at an excitation wavelength of 488 nm and an emission wavelength of 525 nm using a microplate reader (Biotek, USA) equipped with Gen5 software. Untreated cells were used as the blank group. Cells treated with H_2_O_2_ served as the negative control group. Cells treated with 0.3 mg/mL vitamin C (VC) and H_2_O_2_ served as the positive control group. All measurements were repeated three times. The results were presented as the average value ± standard deviation.

### 2.5 ABTS Free Radical Scavenging Assay

ABTS working solution was prepared by mixing 7 mmol/L 2, 2'‐azino‐bis(3‐ethylbenzothiazoline‐6‐sulfonic acid) (ABTS) with 2.45 mmol/L potassium persulfate solution at a 1:1 (v/v) ratio. Then the mixture was incubated in the dark for 12 h and diluted with water till the OD value reached 0.75 ± 0.05 to obtain the ABTS working solution. ABTS working solution was mixed with different concentrations of HA or silybin, and the OD values were measured at a wavelength of 734 nm using a microplate reader (Biotek, USA) equipped with Gen5 software. The tested concentrations of HA solutions were 0.00005%, 0.00025%, 0.0005%, 0.005%, 0.05%, 0.25%, and 0.5% (w/w), respectively. The concentrations of silybin solutions were 0.00005%, 0.000125%, 0.00025%, 0.0005%, 0.00125%, 0.0025% and 0.005% (w/w), respectively. The ABTS scavenging ability *A* was calculated using Equation 1.

(1) $$A=\left(1-\frac{T-T_{0}}{C-C_{0}}\right)\times 100\%$$

Here, T is the OD of sample solutions containing the ABTS working solution; T_0_ is the OD of sample solutions without any ABTS working solution; C is the OD of solvent solutions containing the ABTS working solution; and C_0_ is the OD of solvent solutions without any ABTS working solution. All measurements were repeated three times. The results were presented as the average value ± standard deviation.

### 2.6 Enzyme‐Linked Immunosorbent Assay

For the enzyme‐linked immunosorbent assay (ELISA), HSF cells were seeded in 48‐well plates at a density of 1.2 × 10^5^ cells/cm^2^ and cultured for 24 h. Then, the cells were exposed to PBS containing different concentrations of silybin, HA, or a mixture of silybin and HA with 20 J/cm^2^ UVA irradiation. The tested concentrations of silybin were 0.0001%, 0.005%, and 0.001% (w/w), respectively. The concentrations of HA were 0.01%, 0.05%, and 0.1% (w/w), respectively. The concentrations of silybin and HA in the mixtures were 0.0001% (silybin)+0.01% (HA), 0.005% (silybin)+0.05% (HA), and 0.001% (silybin)+0.1% (HA) (w/w), respectively. Then, the cells were incubated with DMEM containing the same concentrations of silybin, HA, or a mixture of the two and cultured for an additional 24 h. Finally, the cell culture medium was collected and centrifuged at 1000 × g for 20 min to obtain the supernatant. The relative concentrations of Col I and Col III were measured using the Human COL1α1 ELISA Kit (Sangon Biotech, China) and Human COL3 ELISA Kit (Sangon Biotech China) according to the manufacturer's instructions. Cells without any treatment served as the blank group. Cells treated with UV irradiation alone served as the negative control group. All measurements were repeated three times. The results were presented as the average value ± standard deviation.

### 2.7 Statistical Analysis

All data were analyzed using GraphPad Prism for Windows version 9.5.0. Significant differences were assessed using one‐way ANOVA method for multiple comparisons and two‐tailed Student's *t*‐test for pairwise comparisons. A p value less than 0.05 was significant.

## 3 Results and Discussion

### 3.1 Cell Viability Assay

**FIGURE 1:**
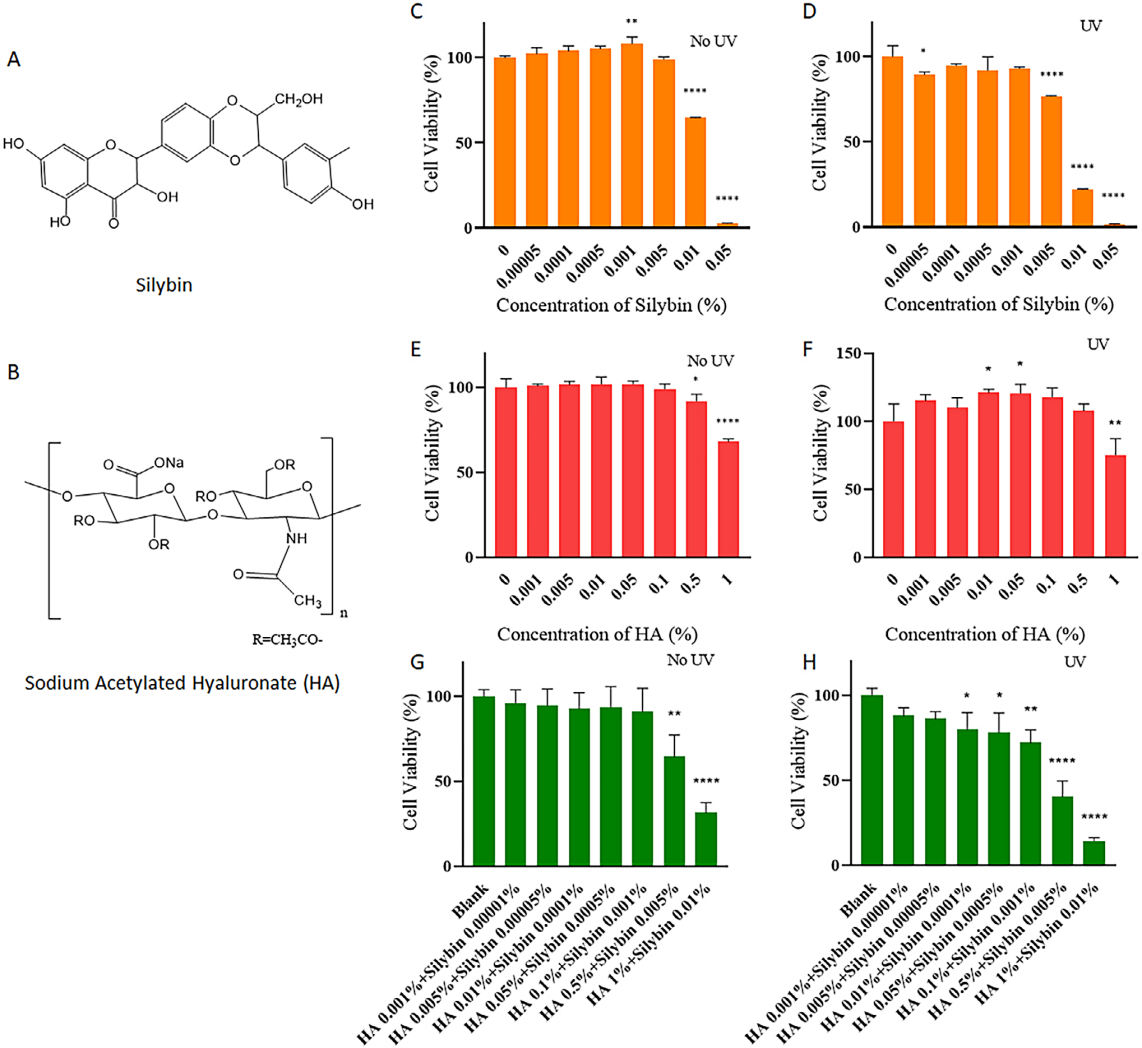
Individual and combined cytotoxicity of HA and silybin. (A) Chemical structure of silybin. (B) Chemical structure of HA. (C‐D) Cell viability after treatment with silybin, without (C) and with (D) UV irradiation. (E‐F) Cell viability after treatment with HA, without (E) and with (F) UV irradiation. (G‐H) Cell viability after treatment with a mixture of HA and silybin, without (G) and with (H) UV irradiation. The p values were calculated using a two‐tailed Student's *t*‐test. (*p ≤ 0.05, **p ≤ 0.01, ***p ≤ 0.001 and ****p ≤ 0.0001 vs. Blank).

Exposure to UV irradiation is inevitable in daily life, but UV‐induced phototoxicity is a major cause of cell death. Therefore, most experiments in our study involved UV treatment. First, we tested the cytotoxicity of silybin and HA in the presence and absence of UV irradiation (Figure 1). Initially, we measured the cytotoxicity of silybin and HA independently. Then, the highest concentrations that did not induce serious toxicity were used to test their combined cytotoxicity.

In the absence of UV irradiation, silybin showed almost no cytotoxicity when its concentration was less than 0.005% (w/w) (Figure 1C). However, when the concentration of silybin exceeded 0.05% (w/w), the cell viability came to ca. 0%. Under UVA irradiation, the cytotoxicity of silybin increased, with obvious cytotoxicity detected at a concentration of 0.005% (w/w) (Figure 1D). When the concentration of silybin exceeded 0.05% (w/w) under these conditions, the cell viability approached 0. This increase in cytotoxicity was due to the combined effects of UV rays and silybin. Nevertheless, the majority of cells were viable when the concentration of silybin was 0.01% (w/w). Therefore, we selected 0.01% (w/w) as the highest concentration of silybin in the combined tests.

HA induced no significant cytotoxicity even when its concentration reached 0.5% (w/w), irrespective of UV exposure (Figures 1E and 1F). In fact, the cell viability remained higher than 50% even when the concentration of HA was 1% (w/w). Therefore, we used 1% (w/w) as the highest concentration of HA in the combined cytotoxicity tests.

Finally, the combined cytotoxicity of silybin and HA was tested at maximum concentrations of 1% (w/w) HA and 0.01% (w/w) silybin, as well as at lower concentrations. When the mixture contained 0.1% (w/w) HA + 0.001% (w/w) silybin, no significant cytotoxicity was observed if UV irradiation was absent (Figure 1G). However, UV irradiation increased the cytotoxicity of this mixture (Figure 1H). Based on these findings, we chose 0.01% (w/w) HA + 0.0001% (w/w) silybin, 0.05% (w/w) HA + 0.0005% (w/w) silybin, and 0.1% (w/w) HA and 0.001% (w/w) silybin to study the combined effects of HA and silybin in subsequent experiments.

The cytotoxicity levels observed in the present study were consistent with the existing literature, since studies have reported that HA is non‐toxic and non‐sensitizing, and can therefore be used safely for all types of skin with no risk of allergic reactions [16]. Notably, our data showed that even in the presence of 1% HA, the treated cells showed satisfactory viability. Any cell death induced after HA treatment was likely a result of osmotic pressure and not the effects of HA itself. Additionally, the combination of silybin and HA exerted similar cytotoxicity as silybin alone. Therefore, HA did not significantly decrease the toxicity of silybin.

### 3.2 Combined Effect of Silybin and HA on mRNA Expression of MMP, Col I and Col III

**FIGURE 2:**
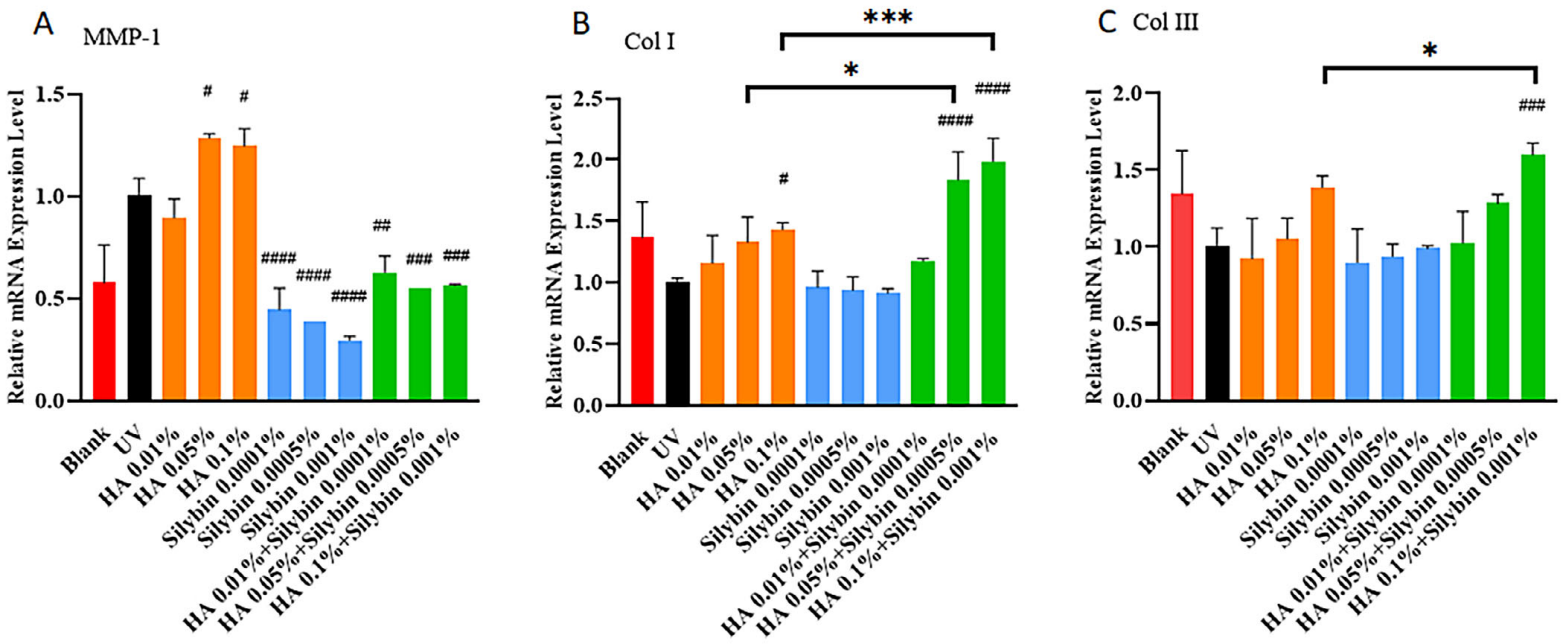
Combined effects of silybin and HA on MMP, Col I and Col III mRNA expression. (A) MMP‐1, (B) Col I, and (C) Col III mRNA expression in the presence of HA, silybin and the mixture of the two. Blank: Cells without treatment. UV: Cells subjected to UV irradiation only. The p values were calculated using the two‐tailed Student's *t*‐test. (*p ≤ 0.05, **p ≤ 0.01, ***p ≤ 0.001 and ****p ≤ 0.0001 vs. Blank; ^#^ *p* ≤ 0.05, ^##^ *p* ≤ 0.01, ^###^ *p* ≤ 0.001 and ^####^ *p* ≤ 0.0001 vs. the UV group).

MMP upregulation following UV exposure is the primary contributor to collagen degradation. The results of the present study indicated that the combination of silybin and HA could effectively inhibit MMP expression (Figure 2A). The mRNA expression of MMP‐1 was significantly suppressed by silybin. The MMP inhibition ability of silybin was so strong that even silybin concentrations of 1 ppm could inhibit MMP‐1 expression by ca. 60%. In contrast, HA was unable to inhibit MMP expression. In fact, after treatment with a high HA concentration, the expression of MMP‐1 was slightly higher than that in UV‐irradiated samples (negative control). This upregulation of MMP was attributed to the combined toxic effects of UV and HA. Although addition of HA of high concentration promoted, rather than inhibited MMP expression as shown in Figure 2A, the promotion effect was so weak that the combination of HA and silybin still showed an MMP inhibition effect. Previously, HA was reported to inhibit MMP expression in H_2_O_2_‐treated HaCaT cells [15]. HA formed a protective layer on the surface of these cells and interacted directly with H_2_O_2_ to attenuate its influence. Consequently, the cells were exposed to less H_2_O_2_‐induced irritation, which alleviated the upregulation of MMP. However, in the present study, HA could not interact directly with UV rays, which limited its impact on MMP‐1 expression. Nevertheless, when UV‐irradiated cells were treated with silybin combined with HA, strong MMP‐1 inhibition was observed.

Interestingly, our findings demonstrated that HA has a strong capacity to promote the expression of collagen mRNAs, but silybin does not (Figures 2B and 2C). These findings were consistent with the understanding that silybin primarily hinders skin aging by inhibiting collagen degradation [11]. Notably, the combination of silybin and HA induced higher collagen expression than HA alone. In previous studies, silybin has been reported to improve cell viability through multiple mechanisms, including the prevention of apoptosis [19], inhibition of DNA breaks [11], and suppression of inflammatory factors [20]. Healthier cells typically show higher collagen expression. This was likely why silybin enhanced the collagen upregulation induced by HA. In summary, the data showed that the combination of silybin and HA was effective at both MMP inhibition and collagen upregulation.

### 3.3 ROS Scavenging Ability Assay

**FIGURE 3:**
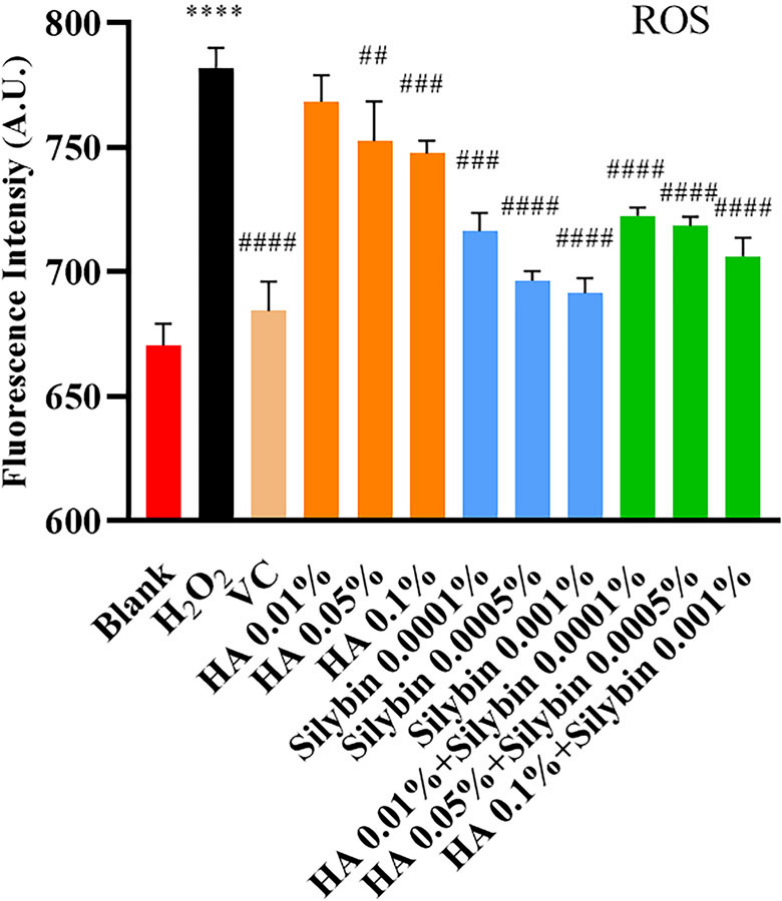
ROS scavenging capacity of silybin and HA. Blank: Untreated cells. Negative control: H_2_O_2_‐treated cells. Positive control: H_2_O_2_‐ and VC‐treated cells. The p values were calculated using the two‐tailed Student's *t*‐test. (*p ≤ 0.05, **p ≤ 0.01, ***p ≤ 0.001 and ****p ≤ 0.0001 vs. Blank; ^#^ *p* ≤ 0.05, ^##^ *p* ≤ 0.01, ^###^ *p* ≤ 0.001 and ^####^ *p* ≤ 0.0001 vs. H_2_O_2_ group).

MMP synthesis is commonly upregulated due to ROS production [21]. To understand the mechanism underlying the effects of silybin and HA on MMP levels, we measured the ROS scavenging capacity of these compounds (Figure 3). After H_2_O_2_ treatment, the ROS levels of cells increased significantly. However, silybin effectively inhibited ROS production even at a concentration of 0.0001% (w/w). Moreover, its inhibition efficiency increased with its concentration. The decrease in ROS production induced by silybin was in line with its inhibition of MMP‐1 mRNA levels. In contrast, HA showed a limited ability to scavenge H_2_O_2_‐induced ROS. We speculate that this may be because HA forms a layer on the surface of cells and only partly protects them from interacting with H_2_O_2_. Nevertheless, the mixture of silybin and HA significantly decreased ROS production, in line with its ability to inhibit MMP expression.

### 3.4 ABTS Scavenging Capacity Assay

**FIGURE 4:**
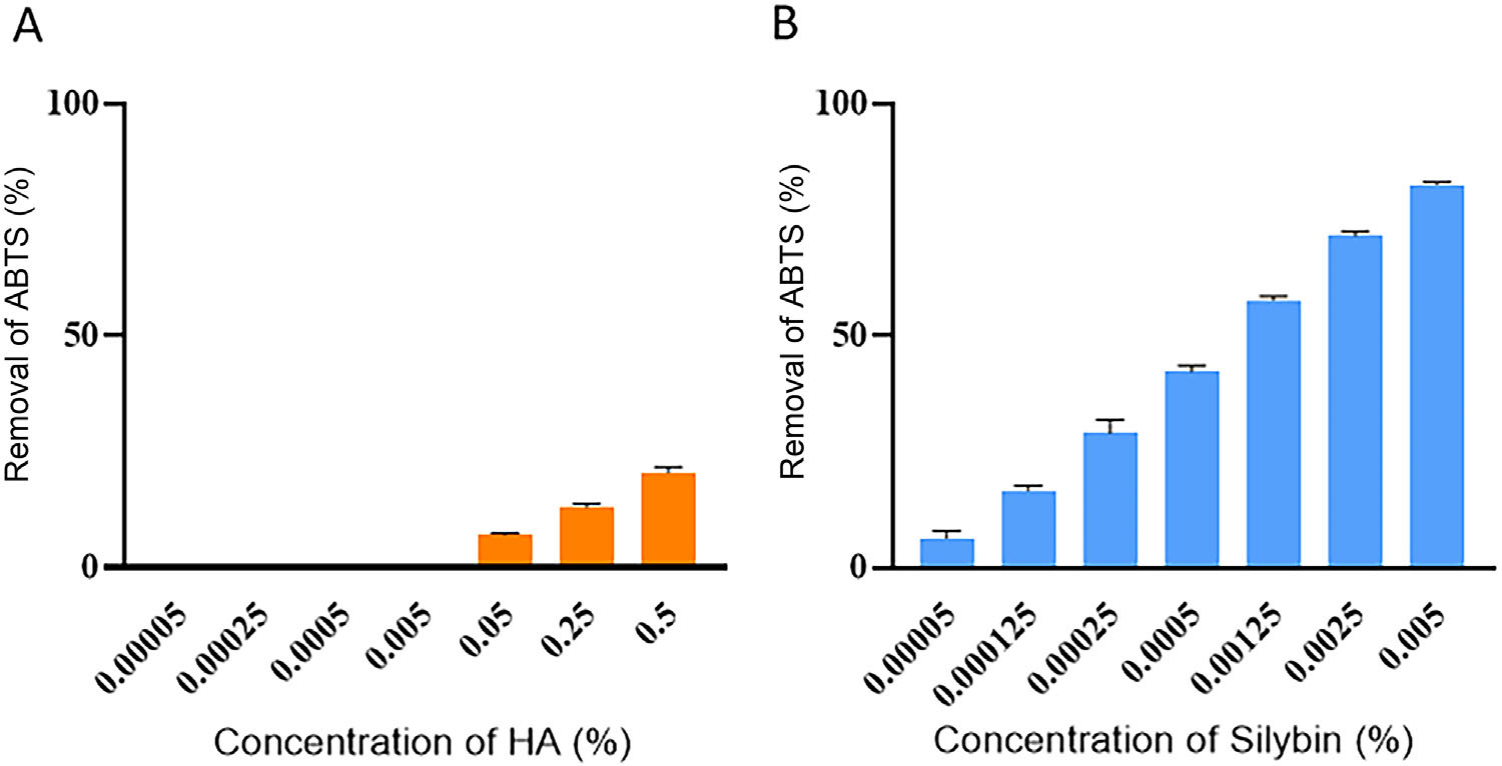
ABTS scavenging ability of (A) HA and (B) silybin.

We measured the reducing ability of silybin and HA based on their ability to scavenge ABTS radicals (Figure 4). The ABTS scavenging capacity of HA was weak, and almost no ABTS was eliminated when the concentration of HA was less than 0.005%. However, the ABTS scavenging capacity of silybin was strong. The removal of ABTS was positively correlated with the concentration of silybin. The chemical structure of silybin contains several phenolic hydroxyl groups, conferring it with powerful reducing ability. The ROS scavenging ability of silybin was consistent with its reducing ability, indicating that this was the mechanism underlying its ROS scavenging effects.

### 3.5 Enhanced Collagen Protein Synthesis

**FIGURE 5:**
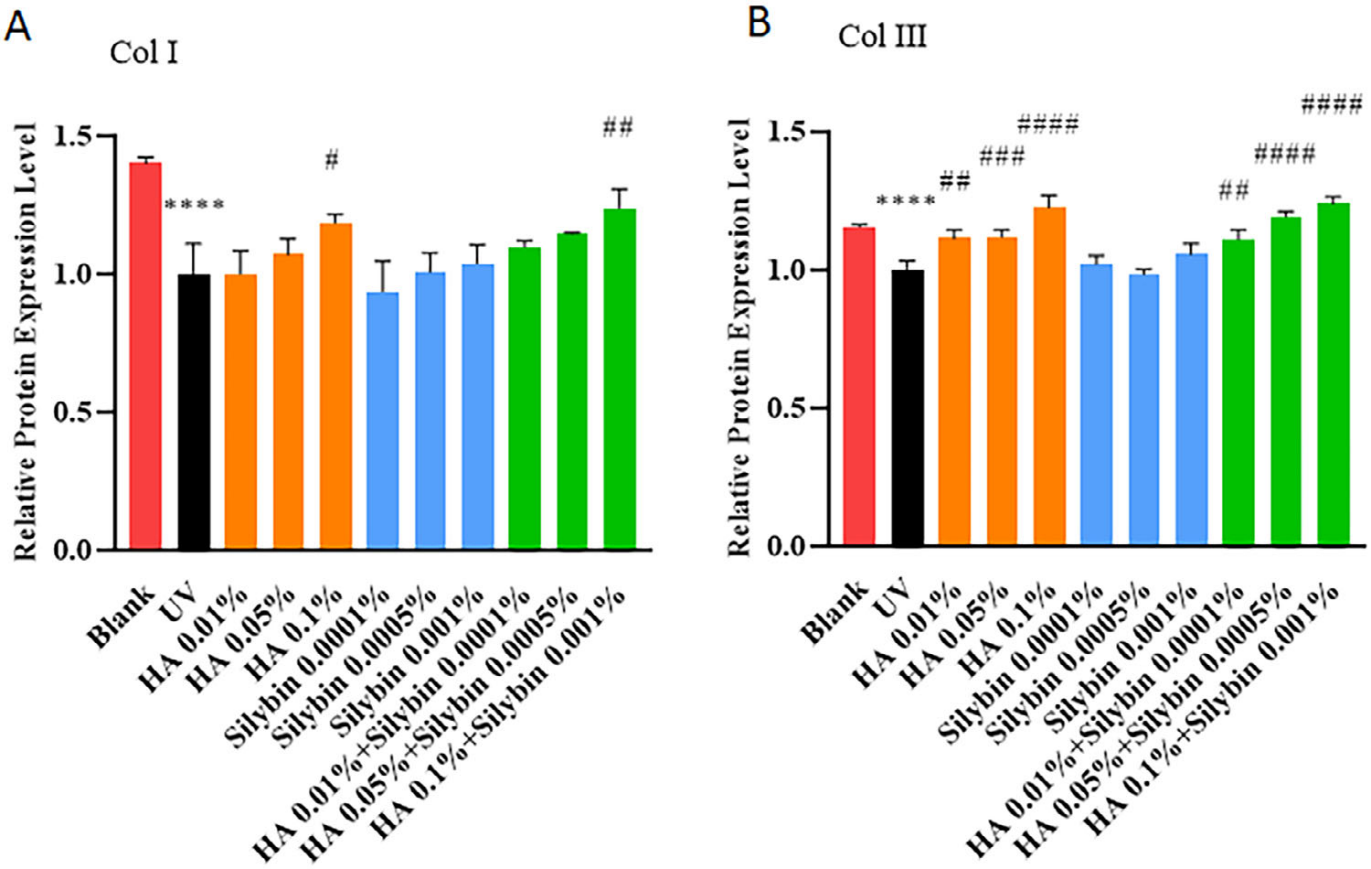
Combined effect of silybin and HA on the synthesis of collagen proteins. (A) Col I and (B) Col III protein synthesis in the presence of HA, silybin, and a mixture of the two compounds. Blank: Untreated cells. UV: Cells exposed to UV irradiation only. The p values were calculated using the two‐tailed Student's *t*‐test. (*p ≤ 0.05, **p ≤ 0.01, ***p ≤ 0.001 and ****p ≤ 0.0001 vs. Blank; ^#^ *p* ≤ 0.05, ^##^ *p* ≤ 0.01, ^###^ *p* ≤ 0.001 and ^####^ *p* ≤ 0.0001 vs. UV group).

Our previous results indicated that silybin protects collagen from MMP‐induced degradation by inhibiting MMP expression, while HA directly promotes collagen synthesis. Notably, using ELISA, we found that the combination of silybin and HA significantly increased the protein levels of Col I and Col III (Figure 5). Interestingly, HA exerted a more obvious effect on these levels than silybin. Notably, our findings showed that HA upregulates the mRNA levels of both Col I and Col III. This upregulation likely led to increased protein translation, enhancing the amount of collagen produced and secreted into the cell culture medium. Meanwhile, silybin inhibited the expression of MMP‐1. However, silybin treatment alone did not produce a statistically significant increase in collagen protein levels compared to the UV‐irradiated control. This observation is consistent with our mRNA data showing that silybin failed to upregulate Col I and III mRNA levels, indicating that silybin does not directly promote collagen synthesis and it primarily protects existing collagen from MMP‐mediated degradation. This represents a limitation of our experimental model, where the protective effect of silybin was not reflected by protein concentrations.

Furthermore, although HA significantly upregulated Col I and Col III mRNA levels, the corresponding increases at the protein level were moderate rather than proportional. This discrepancy between mRNA upregulation and protein accumulation may be attributed to the relatively short treatment duration, post‐transcriptional regulatory mechanisms, and the time required for collagen protein processing, secretion, and extracellular accumulation.

## 4 Discussion

**FIGURE 6:**
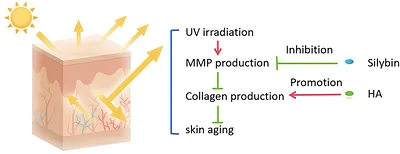
Strategy to achieve combined anti‐aging effect by promoting collagen synthesis and inhibiting collagen degradation simultaneously. HA was used as ingredient to promote collagen synthesis and silybin was used to inhibit collagen degradation in our study.

There are many ingredients beyond HA and silybin that are capable of promoting collagen production. For example, retinal and vitamin C are well known cosmetic ingredients to promote collagen synthesis. However, the combined effect is not always about to occur when randomly choosing two anti‐aging ingredients simultaneously. As we showed in Figure 6, using both ways to promote collagen synthesis and to inhibit collagen degradation simultaneously should be a more efficient strategy to achieve complementary combined effect. In our study, we showed that a combined anti‐aging effect would occur when using HA to promote collagen synthesis and using silybin to inhibit collagen degradation. However, our findings can be expanded to applications that using other two types of ingredients to achieve combined anti‐aging effect: one ingredient to increase collagen production and one to inhibit degradation.

## 5 Conclusion

In this study, we reported the combined effect of silybin and HA on UV‐induced photoaging in a model of HSF cells. Within its toxicity threshold, silybin efficiently inhibited MMP‐1 expression and thus prevented collagen from being degraded. However, silybin did not directly stimulate collagen synthesis. In contrast, HA was effective at directly promoting collagen synthesis, although it did not inhibit, and at higher concentrations slightly elevated MMP‐1 expression. Notably, the inhibitory effect of silybin on MMP expression was attributed to its ROS scavenging and reducing ability. Meanwhile, given its weak ROS scavenging and reducing capacity, HA had a limited effect on MMP expression. Nevertheless, the combination of silybin and HA significantly improved collagen protein levels. Our study elucidates the complementary effects of silybin and HA on collagen homeostasis in fibroblasts: silybin primarily protects existing collagen from MMP‐mediated degradation, while HA promotes de novo collagen synthesis. These findings suggest that silybin and HA could be incorporated together as key ingredients in cosmetic products to protect the skin from UV‐induced photoaging.

## Funding Statement

The authors have nothing to report.

## Conflicts of Interest

Sen Hou, Hanliang Song and Anzhang Li are employees of both Guyu Biotechnology Group Co., Ltd. and Guangzhou Qingnang Biotechnology Co., Ltd. Jianying Zhang is an employee of Guangzhou Zhongkejian Technology Testing Co., Ltd. The employment relationship does not compromise the scientific accuracy, transparency and repeatability of the study. There are no other conflicts of interest.

## Supporting information




Supporting Information: srt70382‐sup‐0001‐tableS1‐S14.docx

## Data Availability

The data that supports the findings of this study are available in the supplementary material of this article.

## References

[1] A. Svobodova and J. Vostalova , “Solar Radiation Induced Skin Damage: Review of Protective and Preventive Options,” International Journal of Radiation Biology 86 (2010): 999–1030. 20807180 10.3109/09553002.2010.501842

[2] T. G. Polefka , T. A. Meyer , P. P. Agin , and R. J. Bianchini , “Effects of Solar Radiation on the Skin,” Journal of Cosmetic Dermatology 11 (2012): 134–143. 22672278 10.1111/j.1473-2165.2012.00614.x

[3] C. López‐Otín , M. A. Blasco , L. Partridge , M. Serrano , and G. Kroemer , “Hallmarks of Aging: An Expanding Universe,” Cell 186 (2023): 243–278. 36599349 10.1016/j.cell.2022.11.001

[4] N. Saewan and A. Jimtaisong , “Natural Products as Photoprotection,” Journal of Cosmetic Dermatology 14 (2015): 47–63. 25582033 10.1111/jocd.12123

[5] B. Anfuso , P. Giraudi , C. Tiribelli , and N. Rosso , “Silybin Modulates Collagen Turnover in an in Vitro Model of NASH,” Molecules 24, no. 7 (2019): 1280, 10.3390/molecules24071280. 30986937 PMC6479571

[6] A. L. Rippa , E. P. Kalabusheva , and E. A. Vorotelyak , “Regeneration of Dermis: Scarring and Cells Involved,” Cells 8, no. 6 (2019): 607, 10.3390/cells8060607. 31216669 PMC6627856

[7] N. Basisty , A. Holtz , and B. Schilling , “Accumulation of ”Old Proteins“ and the Critical Need for MS‐Based Protein Turnover Measurements in Aging and Longevity,” John Wiley & Sons, Ltd 20, no. 5–6 (2020): 1800403, 10.1002/pmic.201800403. PMC7015777 31408259

[8] K. K. Dong , N. Damaghi , S. D. Picart , N. G. Markova , and D. B. Yarosh , “UV‐Induced DNA Damage Initiates Release of MMP‐1 in Human Skin,” Experimental Dermatology 17 (2008): 1037–1044. 18459971 10.1111/j.1600-0625.2008.00747.x

[9] T. Quan and G. J. Fisher , “Role of Age‐Associated Alterations of the Dermal Extracellular Matrix Microenvironment in Human Skin Aging: A Mini‐Review,” Gerontology 61 (2015): 427–434. 25660807 10.1159/000371708 PMC4524793

[10] J. Varani , M. K. Dame , L. Rittie , et al., “Decreased Collagen Production in Chronologically Aged Skin: Roles of Age‐Dependent Alteration in Fibroblast Function and Defective Mechanical Stimulation,” American Journal of Pathology 168 (2006): 1861–1868. 16723701 10.2353/ajpath.2006.051302 PMC1606623

[11] A. Rajnochova Svobodova , E. Gabrielova , L. Michaelides , et al., “UVA‐Photoprotective Potential of Silymarin and Silybin,” Archives of Dermatological Research 310 (2018): 413–424. 29564550 10.1007/s00403-018-1828-6

[12] A. Svobodova , Z. Adela , D. Walterova , and J. Vostalova , “Flavonolignans From Silybum Marianum Moderate UVA‐Induced Oxidative Damage to HaCaT Keratinocytes,” Journal of Dermatological Science 48 (2007): 213–224. 17689055 10.1016/j.jdermsci.2007.06.008

[13] J. Fraser , T. Laurent , and U. Laurent , “Hyaluronan: Its Nature, Distribution, Functions and Turnover,” Journal of Internal Medicine 242 (1997): 27–33. 9260563 10.1046/j.1365-2796.1997.00170.x

[14] M. Essendoubi , C. Gobinet , R. Reynaud , J. F. Angiboust , M. Manfait , and O. Piot , “Human Skin Penetration of Hyaluronic Acid of Different Molecular Weights as Probed by Raman Spectroscopy,” Skin Research & Technology 22 (2016): 55–62. 25877232 10.1111/srt.12228

[15] M. Meunier , A. Scandolera , E. Chapuis , et al., “The Anti‐Wrinkles Properties of Sodium Acetylated Hyaluronate,” Journal of cosmetic dermatology 21 (2022): 2749–2762. 34708918 10.1111/jocd.14539 PMC9543187

[16] S. N. A. Bukhari , N. L. Roswandi , M. Waqas , et al., “Hyaluronic Acid, a Promising Skin Rejuvenating Biomedicine: A Review of Recent Updates and Pre‐Clinical and Clinical Investigations on Cosmetic and Nutricosmetic Effects,” International Journal of Biological Macromolecules: Structure, Function and Interactions 120 (2018): 1682–1695. 10.1016/j.ijbiomac.2018.09.188 30287361

[17] S. Gariboldi , M. Palazzo , L. Zanobbio , et al., “Low Molecular Weight Hyaluronic Acid Increases the Self‐Defense of Skin Epithelium by Induction of Beta‐Defensin 2 via TLR2 and TLR4,” Journal of Immunology 43 (2008): 310–310. 10.4049/jimmunol.181.3.2103 18641349

[18] T. Murakami , S. Otsuki , Y. Okamoto , et al., “Hyaluronic Acid Promotes Proliferation and Migration of Human Meniscus Cells via a CD44‐Dependent Mechanism,” Taylor & Francis 60 (2019): 117–127. 10.1080/03008207.2018.1465053 29658360

[19] S. Dhanalakshmi , G. U. Mallikarjuna , R. P. Singh , and R. Agarwal , “Dual Efficacy of Silibinin in Protecting or Enhancing Ultraviolet B Radiation‐Caused Apoptosis in HaCaT Human Immortalized Keratinocytes,” Carcinogenesis 25 (2004): 99–106. 14555614 10.1093/carcin/bgg188

[20] S. Kitajima and K. Yamaguchi , “Silybin From Silybum Marianum Seeds Inhibits Confluent‐Induced Keratinocytes Differentiation as Effectively as Retinoic Acid Without Inducing Inflammatory Cytokine,” Journal of Clinical Biochemistry & Nutrition 45 (2009): 178–184. 19794926 10.3164/jcbn.09-20 PMC2735630

[21] K. I. M. Jung‐Ae , A. H. N. Chang‐Suk , E. O. M. Byulnim , et al., “Effects of Carboxymethyl‐Chitosan and ‐Chitin on MMP Inhibition Related With ROS Scavenging in HT1080 human Fibrosarcoma Cells,” The Korean Society for Biotechnology and Bioengineering 4 (2009): 209–209.

