# Supplementary material for: Combined Effect of Silybin and Sodium Acetylated Hyaluronate on UV‐Induced Photoaging
Source: Skin Res Technol. 2026 Sep 12;32(9):e70382. doi: 10.1111/srt.70382 (PMC13570327; doi:10.1111/srt.70382)
Supplement: Supplementary file 1 [file SRT-32-e70382-s001.docx]

Supporting Information

Combined Effect of Silybin and Sodium Acetylated Hyaluronate on UV-Induced Photoaging

Sen Hou^a,b,*^, Hanliang Song^a,b^, Jianying Zhang^c^, Anzhang Li^a,b,*^

^a^ Guyu Biotechnology Group Co., Ltd. Guangzhou, Guangdong 510000, China

^b^ Guangzhou Qingnang Biotechnology Co., Ltd. Guangzhou, Guangdong 510000, China

^c^ Guangzhou Zhongkejian Technology Testing Co., Ltd. Guangzhou, Guangdong 510000, China

^*^Corresponding author, E-mail address: [housen@guyu.com.cn](mailto:housen@guyu.com.cn) (S. Hou), [lianzhang@guyu.com.cn](mailto:lianzhang@guyu.com.cn) (A. Li)

**Table of Contents**

Table S1: Original data for the cell viability after treatment with silybin, without UV irradiation shown in Figure 1C.

Table S2: Original data for the cell viability after treatment with silybin, with UV irradiation shown in Figure 1D.

Table S3: Original data for the cell viability after treatment with HA, without UV irradiation shown in Figure 1E.

Table S4: Original data for the cell viability after treatment with HA, with UV irradiation shown in Figure 1F.

Table S5: Original data for the cell viability after treatment with a mixture of HA and silybin, without UV irradiation shown in Figure 1G.

Table S6: Original data for the cell viability after treatment with a mixture of HA and silybin, with UV irradiation shown in Figure 1H.

Table S7: Original data for the MMP-1 mRNA expression in the presence of HA, silybin and the mixture of the two shown in Figure 2A.

Table S8: Original data for the Col I mRNA expression in the presence of HA, silybin and the mixture of the two shown in Figure 2B.

Table S9: Original data for the Col III mRNA expression in the presence of HA, silybin and the mixture of the two shown in Figure 2C.

Table S10: Original data for the ROS scavenging capacity of silybin and HA shown in Figure 3.

Table S11: Original data for the ABTS scavenging ability of HA shown in Figure 4A.

Table S12: Original data for the ABTS scavenging ability of silybin shown in Figure 4B.

Table S13: Original data for the Col I protein expression in the presence of HA, silybin and the mixture of the two shown in Figure 5A.

Table S14: Original data for the Col III protein expression in the presence of HA, silybin and the mixture of the two shown in Figure 5B.

Table S1: Original data for the cell viability after treatment with silybin, without UV irradiation shown in Figure 1C.

| Concentration (w/w %) | 0 | 0.00005 | 0.0001 | 0.0005 | 0.001 | 0.005 | 0.01 | 0.05 |
| --- | --- | --- | --- | --- | --- | --- | --- | --- |
| Cell Viability (%) | 99.24127466 | 98.63429439 | 104.2488619 | 103.9453718 | 104.7040971 | 97.87556904 | 64.49165402 | 2.731411229 |
|  | 100.9104704 | 102.124431 | 106.676783 | 106.9802731 | 107.8907436 | 100.6069803 | 64.49165402 | 2.427921093 |
|  | 99.84825493 | 105.9180577 | 100.7587253 | 104.400607 | 112.1396055 | 98.63429439 | 64.94688923 | 2.731411229 |

Table S2: Original data for the cell viability after treatment with silybin, with UV irradiation shown in Figure 1D.

| Concentration (w/w %) | 0 | 0.00005 | 0.0001 | 0.0005 | 0.001 | 0.005 | 0.01 | 0.05 |
| --- | --- | --- | --- | --- | --- | --- | --- | --- |
| Cell Viability (%) | 98.02538787 | 89.42172073 | 94.35825106 | 99.57686883 | 92.8067701 | 77.15091678 | 21.86177715 | 1.551480959 |
|  | 107.0521862 | 88.0112835 | 95.76868829 | 92.2425952 | 92.66572638 | 76.58674189 | 22.00282087 | 1.128349788 |
|  | 94.92242595 | 90.97320169 | 93.79407616 | 84.06205924 | 93.93511989 | 76.16361072 | 22.42595205 | 1.833568406 |

Table S3: Original data for the cell viability after treatment with HA, without UV irradiation shown in Figure 1E.

| Concentration (w/w %) | 0 | 0.001 | 0.005 | 0.01 | 0.05 | 0.1 | 0.5 | 1 |
| --- | --- | --- | --- | --- | --- | --- | --- | --- |
| Cell Viability (%) | 100.0495535 | 101.98216 | 102.7254703 | 106.5906833 | 101.98216 | 100.1982156 | 95.88701637 | 69.57383514 |
|  | 104.9554009 | 100.3468776 | 99.45490535 | 99.45490535 | 103.6174425 | 101.0901878 | 91.87314127 | 66.30327024 |
|  | 94.99504413 | 100.3468776 | 102.7254703 | 99.75222944 | 100.1982156 | 95.58969229 | 88.00792821 | 68.38453881 |

Table S4: Original data for the cell viability after treatment with HA, with UV irradiation shown in Figure 1F.

| Concentration (w/w %) | 0 | 0.001 | 0.005 | 0.01 | 0.05 | 0.1 | 0.5 | 1 |
| --- | --- | --- | --- | --- | --- | --- | --- | --- |
| Cell Viability (%) | 109.7746908 | 114.6874471 | 116.8897171 | 123.6659326 | 126.3764188 | 122.8189056 | 112.8239878 | 85.04150432 |
|  | 85.88853126 | 119.7696087 | 110.6217178 | 120.6166356 | 121.8024733 | 120.2778248 | 102.6596646 | 78.60409961 |
|  | 104.3537184 | 110.9605285 | 102.9984754 | 118.7531764 | 113.8404201 | 109.9440962 | 107.4030154 | 61.83296629 |

Table S5: Original data for the cell viability after treatment with a mixture of HA and silybin, without UV irradiation shown in Figure 1G.

| Silybin (w/w %) | 0 | 0.00001 | 0.00005 | 0.0001 | 0.0005 | 0.001 | 0.005 | 0.01 |
| --- | --- | --- | --- | --- | --- | --- | --- | --- |
| HA (w/w %) | 0 | 0.001 | 0.005 | 0.01 | 0.05 | 0.1 | 0.5 | 1 |
| Cell Viability (%) | 95.71906995 | 86.88963792 | 83.47826645 | 82.4749219 | 80.66890172 | 75.25084115 | 49.96655853 | 24.88294481 |
|  | 103.5451574 | 100.9364616 | 99.93311705 | 95.91973886 | 94.91639431 | 96.52174559 | 69.43144277 | 33.71237684 |
|  | 100.7357927 | 100.133786 | 100.3344549 | 99.93311705 | 104.9498398 | 100.9364616 | 73.84615879 | 36.12040375 |

Table S6: Original data for the cell viability after treatment with a mixture of HA and silybin, with UV irradiation shown in Figure 1H.

| Silybin (w/w %) | 0 | 0.00001 | 0.00005 | 0.0001 | 0.0005 | 0.001 | 0.005 | 0.01 |
| --- | --- | --- | --- | --- | --- | --- | --- | --- |
| HA (w/w %) | 0 | 0.001 | 0.005 | 0.01 | 0.05 | 0.1 | 0.5 | 1 |
| Cell Viability (%) | 95.50561798 | 82.47191011 | 81.34831461 | 68.76404494 | 65.39325843 | 66.51685393 | 34.60674157 | 13.25842697 |
|  | 100.6741573 | 89.66292135 | 88.76404494 | 82.47191011 | 82.02247191 | 68.53932584 | 35.28089888 | 13.03370787 |
|  | 103.8202247 | 91.46067416 | 88.31460674 | 88.31460674 | 86.96629213 | 80.6741573 | 51.01123596 | 16.40449438 |

Table S7: Original data for the MMP-1 mRNA expression in the presence of HA, silybin and the mixture of the two shown in Figure 2A.

|  | Blank | UV | HA 0.01% | HA 0.05% | HA 0.1% | Silybin 0.0001% | Silybin 0.0005% | Silybin 0.001% | HA 0.01%+  Silybin 0.0001% | HA 0.05%+  Silybin 0.0005% | HA 0.1%+  Silybin 0.001% |
| --- | --- | --- | --- | --- | --- | --- | --- | --- | --- | --- | --- |
| Relative RNA  Expression level | 0.48 | 0.95 | 0.96 | 1.27 | 1.29 | 0.52 | 0.39 | 0.31 | 0.7 | 0.55 | 0.57 |
|  | 0.79 | 1.1 | 0.83 | 1.3 | 1.3 | 0.37 | 0.39 | 0.28 | 0.54 | 0.55 | 0.56 |
|  | 0.46 | 0.96 | / | / | 1.15 | / | / | / | 0.64 | / | / |

Table S8: Original data for the Col I mRNA expression in the presence of HA, silybin and the mixture of the two shown in Figure 2B.

|  | Blank | UV | HA 0.01% | HA 0.05% | HA 0.1% | Silybin 0.0001% | Silybin 0.0005% | Silybin 0.001% | HA 0.01%+  Silybin 0.0001% | HA 0.05%+  Silybin 0.0005% | HA 0.1%+  Silybin 0.001% |
| --- | --- | --- | --- | --- | --- | --- | --- | --- | --- | --- | --- |
| Relative RNA  Expression level | 1.7 | 1.024759334 | 1.240950499 | 1.131357028 | 1.440440722 | 0.949694985 | 0.84727386 | 0.957222144 | 1.148081475 | 1.712201293 | 1.778585133 |
|  | 1.2 | 0.957314745 | 1.331962823 | 1.310695897 | 1.482391277 | 1.098429144 | 0.904824455 | 0.895695229 | 1.18515271 | 2.101667785 | 2.038888618 |
|  | 1.2 | 1.019350097 | 0.907937789 | 1.539229098 | 1.371237355 | 0.833639947 | 1.05622132 | 0.89641081 | 1.186661203 | 1.696859601 | 2.144524965 |

Table S9: Original data for the Col III mRNA expression in the presence of HA, silybin and the mixture of the two shown in Figure 2C.

|  | Blank | UV | HA 0.01% | HA 0.05% | HA 0.1% | Silybin 0.0001% | Silybin 0.0005% | Silybin 0.001% | HA 0.01%+  Silybin 0.0001% | HA 0.05%+  Silybin 0.0005% | HA 0.1%+  Silybin 0.001% |
| --- | --- | --- | --- | --- | --- | --- | --- | --- | --- | --- | --- |
| Relative RNA  Expression level | 1.67 | 1.07817921 | 1.19562049 | 0.901639029 | 1.330399145 | 0.860133248 | 0.956393015 | 1.007395593 | 0.796822554 | 1.327849348 | 1.630557681 |
|  | 1.17 | 1.069327254 | 0.671470902 | 1.136453431 | 1.474390076 | 1.133332503 | 0.851308409 | 0.993767146 | 1.200461189 | 1.218495607 | 1.519814467 |
|  | 1.2 | 0.867358045 | 0.903240435 | 1.124874362 | 1.356802247 | 0.707289318 | 1.008798104 | 0.969074213 | 1.080375102 | 1.305160347 | 1.658448718 |

Table S10: Original data for the ROS scavenging capacity of silybin and HA shown in Figure 3.

|  | Blank | H_2_O_2_ | VC | HA 0.01% | HA 0.05% | HA 0.1% | Silybin 0.0001% | Silybin 0.0005% | Silybin 0.001% | HA 0.01%+  Silybin 0.0001% | HA 0.05%+  Silybin 0.0005% | HA 0.1%+  Silybin 0.001% |
| --- | --- | --- | --- | --- | --- | --- | --- | --- | --- | --- | --- | --- |
| Fluorescence Intensity  (A.U.) | 671 | 774 | 671 | 756 | 771 | 742 | 719 | 695 | 685 | 725 | 715 | 698 |
|  | 662 | 790 | 688 | 775 | 742 | 749 | 722 | 694 | 696 | 724 | 719 | 709 |
|  | 679 | 782 | 694 | 774 | 745 | 752 | 708 | 701 | 694 | 719 | 722 | 712 |

Table S11: Original data for the ABTS scavenging ability of HA shown in Figure 4A.

| HA (%) | 0.00005 | 0.00025 | 0.0005 | 0.005 | 0.05 | 0.25 | 0.5 |
| --- | --- | --- | --- | --- | --- | --- | --- |
| Removal of ABTS (%) | 0.364963504 | 0.091240876 | -1.003649635 | -0.18 | 6.660583942 | 12.1350365 | 19.25182482 |
|  | -0.729927007 | -1.277372263 | -0.729927007 | -0.18 | 7.481751825 | 13.77737226 | 21.71532847 |
|  | -1.003649635 | -0.45620438 | -1.003649635 | 0.36 | 6.934306569 | 12.95620438 | 20.34671533 |

Table S12: Original data for the ABTS scavenging ability of silybin shown in Figure 4B.

| Silybin (%) | 0.00005 | 0.000125 | 0.00025 | 0.0005 | 0.00125 | 0.0025 | 0.005 |
| --- | --- | --- | --- | --- | --- | --- | --- |
| Removal of ABTS (%) | 4.981203008 | 17.38721805 | 26.40977444 | 42.76315789 | 57.42481203 | 70.39473684 | 82.51879699 |
|  | 6.109022556 | 15.41353383 | 28.38345865 | 40.78947368 | 56.86090226 | 71.52255639 | 81.95488722 |
|  | 8.082706767 | 17.10526316 | 32.04887218 | 43.04511278 | 58.55263158 | 72.36842105 | 83.08270677 |

Table S13: Original data for the Col I protein expression in the presence of HA, silybin and the mixture of the two shown in Figure 5A.

|  | Blank | UV | HA 0.01% | HA 0.05% | HA 0.1% | Silybin 0.0001% | Silybin 0.0005% | Silybin 0.001% | HA 0.01%+  Silybin 0.0001% | HA 0.05%+  Silybin 0.0005% | HA 0.1%+  Silybin 0.001% |
| --- | --- | --- | --- | --- | --- | --- | --- | --- | --- | --- | --- |
| Relative Protein  Expression level | 1.4251005 | 0.8859794 | 0.9618423 | 1.1016461 | 1.1556252 | 0.9034219 | 1.0830978 | 1.0623665 | 1.1086315 | 1.1497095 | 1.1782148 |
|  | 1.3810966 | 1.1051369 | 0.9452056 | 1.103973 | 1.2178755 | 1.0577785 | 0.9507423 | 0.9629548 | 1.0715635 | 1.1438061 | 1.2154562 |
|  | 1.3965456 | 1.0088836 | 1.0958371 | 1.0043737 | 1.1794083 | 0.8459654 | 0.9863978 | 1.0888822 | 1.1109632 | 1.1426269 | 1.3151479 |

Table S14: Original data for the Col III protein expression in the presence of HA, silybin and the mixture of the two shown in Figure 5B.

|  | Blank | UV | HA 0.01% | HA 0.05% | HA 0.1% | Silybin 0.0001% | Silybin 0.0005% | Silybin 0.001% | HA 0.01%+  Silybin 0.0001% | HA 0.05%+  Silybin 0.0005% | HA 0.1%+  Silybin 0.001% |
| --- | --- | --- | --- | --- | --- | --- | --- | --- | --- | --- | --- |
| Relative Protein  Expression level | 1.15235801 | 1.00947736 | 1.13587196 | 1.09656593 | 1.27632998 | 1.05440082 | 0.96821492 | 1.07056716 | 1.14575626 | 1.19551887 | 1.20888641 |
|  | 1.14905561 | 1.02546362 | 1.12272951 | 1.14575626 | 1.20219768 | 0.99673473 | 1.0031007 | 1.08354633 | 1.08029734 | 1.20888641 | 1.24248115 |
|  | 1.1622797 | 0.96505902 | 1.0770514 | 1.1096275 | 1.19551887 | 1.00628789 | 0.97769787 | 1.00947736 | 1.10309137 | 1.17222505 | 1.2593739 |
